# The Natural History of Symptomatic Fractures in Children and Adolescents with Osteogenesis Imperfecta Type 1: A Cohort Study from Western Australia

**DOI:** 10.1002/jbm4.10782

**Published:** 2023-06-21

**Authors:** Kiranjit K Joshi, Aris Siafarikas, Richard Prince

**Affiliations:** ^1^ University of Western Australia Perth WA Australia; ^2^ Perth Children's Hospital Department of Endocrinology and Diabetes Perth WA Australia; ^3^ Western Australian Bone Research Collaboration Perth WA Australia; ^4^ Institute for Health Research University of Notre Dame Australia Fremantle WA Australia; ^5^ Exercise Medicine Research Institute Edith Cowan University Perth WA Australia

**Keywords:** CHILDREN, FRACTURE RISK, OSTEOGENESIS IMPERFECTA TYPE 1, RECURRENT FRACTURES

## Abstract

The fracture experience of children and adolescents with osteogenesis imperfecta (OI) type 1 is not well described in the literature. We present data on symptomatic long bones and axial skeleton fractures of all patients aged 0 to 18 years with OI type 1 seen at a specialized bone clinic in Western Australia in the period 2008 to 2020 using a retrospective chart review method. The cohort consisted of 44 patients (21 males, 23 females). Median (interquartile range [IQR]) age was 11.3 (6.2 to 17) years, giving a total of 520 patient‐years in the study during which 197 fractures were experienced. The mean fracture rate was 379 fractures per 1000 patient‐years (95% confidence interval [CI]: 310 to 440); however, the experience for fractures varied from ≤1 fracture in 23% (*n* = 10) to two to 20 in 77% (*n* = 34) of the cohort. Twenty‐one patients (48.5%) received bisphosphonates during the study period. In logistic regression, age, but not sex or family history of OI, was a significant predictor of fracture risk. The highest total fracture rate was observed in the age group 0 to <3 years at 469 fractures/1000 patient‐years, which declined to 140 fractures/1000 patient‐years in the age group 15 to 18 years. The lower limbs were the site of 49.7% of all fractures. The highest rate for lower limb fracture was in the age group 0 to <3 years at 331 fractures/1000 patient‐years, decreasing to 0 fractures/1000 patient‐years in the age group 15 to 18 years. Upper limb fracture rates increased from 100 fractures/1000 patient‐years in the 0 to <3 years age group to 307 fractures/1000 patient‐years in the 9 to <12 years age group and then declining to 70 fractures/1000 years in the 15 to 18 years age group. In pediatric patients with OI type 1, fracture risk is highest in early life, especially in the lower limbs. Multidisciplinary care of children with OI should have a particular focus on strategies to prevent these fractures. © 2023 The Authors. *JBMR Plus* published by Wiley Periodicals LLC on behalf of American Society for Bone and Mineral Research.

## Graphical abstract

In pediatric patients with osteogenesis imperfecta (OI) type 1, fracture risk is highest in early life, especially in the lower limbs. Multidisciplinary care of children with OI should have a particular focus on strategies to prevent these fractures.

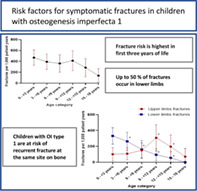

## Introduction

Osteogenesis imperfecta (OI) is a heterogeneous group of inherited connective tissue disorder most commonly caused by mutations in the type 1 collagen genes COL1A1 and COL1A2 characterized by increased propensity for fractures.^[^ ^1^ ^]^ It is the most common genetic condition causing increased bone fragility.

Despite the significant morbidity suffered by OI patients, there are few detailed clinical studies of the natural history of fractures of individual children with OI. A study from Scotland of older adults (aged 60 ± 10 years) with OI reported high fracture rates in their childhood,^[^ ^2^ ^]^ where the fracture rates were determined on memory recall based on questionnaires. More recently, a Danish study examined the fracture rate in a population‐based cohort of all OI patients, including children, and found high fracture rates in the age group of 0 to 19 years.^[^ ^3^ ^]^ Two studies have reported on fracture incidence in OI^[^ ^4^, ^5^ ^]^; however, these studies do not capture the natural history of fractures over time.

The aim of this longitudinal cohort study was to characterize the fractures and fracture prevalence in patients with OI type 1 in childhood at different stages of bone growth. To improve the prognostic advice to patients and parents, we characterized the fracture phenotype and risk factors for fractures in OI in the most common OI subtype, OI type 1 excluding patients with other types of OI given their different prognoses.

## Methods

### Study design and patient cohort

We report a retrospective longitudinal cohort study of pediatric patients age ≤18 years with OI type 1 who attended bone clinics in the Department of Endocrinology at Perth Children's Hospital, from 2008 to 2020. Demographic, clinical, and fracture data were extracted from the West Australia (WA) pediatric endocrine registry and database, the Endocrine Clinical Management System (ECMS) at Perth Children's hospital (PCH), as well as patient records. Data were collected from the time of diagnosis of OI till the end of the study year (2020) or till 18 years of age, when the patients are transitioned to adult services. The patients and their parents gave written consent to include their data in the database for use in audit and research, which is approved by the ethics committee of the WA Child and Adolescent Health Services (Approval No. 1517EP). Perth Children's Hospital is the only tertiary center in WA for pediatric endocrinology. Patients there are likely to represent the majority of the cohort of pediatric OI of WA requiring expert medical help.

Patients were diagnosed with OI type 1 using the Sillence classification^[^ ^6^ ^]^ by experienced clinicians or by medical geneticists based on clinical features, that is, history of fractures, gray‐blue sclerae, hypermobile joints, dentinogenesis imperfecta, and family history of OI, and with genetic testing where indicated.

### Variables studied

#### Demographics and clinical data

For each patient with OI type 1, age at the time of study, sex, and family history of OI were recorded. Clinical characteristics such as gray‐blue sclerae, dentinogenesis imperfecta, hypermobile joints, history of wormian bones, hearing deficit, and ambulatory status at the time of the study were obtained from written records where available.

#### Fracture identification

The clinical record of each patient was reviewed for evidence of symptomatic long bone and axial skeleton fractures as identified by radiological report or confirmed in writing by a clinician where X‐ray was not available. Fractures were classified by patient age at the time of fracture, the long bone or appendicular bone, and site of fracture on the bone. Short bone fractures of the hands and feet were not included in the fracture count. The event associated with each facture was recorded. If more than one fracture occurred at one event, each bone fracture was recorded as a separate fracture.

#### Vertebral fractures

Spine radiology was obtained only if there were clinical concerns, such as back pain or history of trauma to the back. At each clinic visit patients were asked about their back pain history. Vertebral fractures were analyzed by pediatric radiologists and were reported as mild, moderate, or severe based on the loss of vertebral height.

#### Patient‐years and fracture rates

Each year of age for each patient in the study was counted as a patient‐year of the study. The patient‐years of the cohort were divided into six age categories, 0 to < 3 years, 3 to < 6 years, 6 to < 9 years, 9 to < 12 years, 12 to < 15 years, and 15–18 years, and fracture rates (number of fractures per 1000 patient‐years) were calculated for each age category. If children were born with fractures, whether developed antenatally or sustained during birth, the fracture was counted in the first year of life.

#### Same site recurrent fractures

Children who developed radiologically confirmed fractures at the same anatomical site as a previously healed fracture were considered to have the same site fracture.

#### Bisphosphonates

The time of commencement and cessation of intravenous bisphosphonate, but not the pharmaceutical type or dose, was recorded for each patient. In general, the dose was calculated at 0.5 mg/kg/dose for pamidronate and 0.05 mg/kg/dose for zoledronate. For the statistical analysis of fracture events, bisphosphonate use was classified as patients who did not receive any bisphosphonate during the study period and those who received bisphosphonates. Among the patients who received bisphosphonates, fracture events were categorized as occurring in the patient‐years before, during, and after bisphosphonate therapy.

### Statistical analysis

Descriptive statistics were used to summarize the demographics and clinical characteristics of the cohort. Data are presented as mean and 95% confidence interval (CI) or ± SD or median (interquartile range [IQR]) for continuous variables. For categorical variables, data are presented as numbers and percentages. Statistical analysis was done using SPSS (version 22.0). Pearson chi‐squared test was used to analyze differences in proportions. Spearman's rho correlation was used to analyze correlations between variables, such as number of fractures with age at fracture. For independent samples, a Kruskall‐Wallis test was used to analyze associations between continuous and categorical variables. Logistic regression was performed to ascertain the effects of age, sex, family history of OI, and bisphosphonate use on the likelihood of having a fracture. An alpha of 0.05 in a two‐tailed test was set as the cut‐off for statistical significance.

## Results

### Cohort

Forty‐four children with OI type 1, aged 0 to 18 years (21 male, 23 female), mean age at time of study (11.3 [95% CI: 9.8 to 13.1] years) was included in the study. The demographic and clinical characteristics of the patients are presented in Table 1.

**Table 1:** Demographics and fracture characteristics of cohort

Demographics	
Total patients, *n*	44
Genetic diagnosis	8
Diagnosis on bone biopsy	1
Sex, *n* (%)	
Male	21 (48)
Female	23 (52)
Total patient‐years in study, *n*	520
Median age of cohort, years (IQR)	11.3 (6.2–17)
Age category at time of study, *n* (%)	
0 to <3 years	3 (6.8)
3 to <6 years	7 (16)
6 to <9 years	7 (16)
9 to <12 years	6 (13.6)
12 to <15 years	6 (13.6)
15 to 18 years	15 (34)
Family history of OI, *n* (%)	
Positive	35 (79.5)
Negative	8 (18.2)
Unknown	1 (2.2)
Extra skeletal clinical characteristics	
	Yes, No, Unknown (*n*)
Wormian bones	9, 7, 28
Hypermobile joints	22, 6, 16
Dentinogenesis imperfecta	6, 24, 14
Gray‐blue sclerae	36, 2, 6
Hearing deficit	1, 25, 18
Fractures	
Median age at first fracture, years (IQR)	1.4 (0.2–2.6)
Median age for all fractures, years (IQR)	5.0 (2.5–9.0)
Median fractures per patient (IQR)	3 (2–6)
Total fractured bones, *n*	197
Lower limb fractures, *n* (%)	98 (49.7)
Upper limb fractures, *n* (%)	80 (40.6)
Axial fractures, *n* (%)	19 (9.7)
Fracture rate (fractures/1000 patient‐years, 95% CI)	379 (310–440)
Patients with one or fewer fractures, *n* (%)	10 (22.7)
Patients with more than one fracture, *n* (%)	34 (77.3)
Patient‐years with ≥1 fracture, *n* (%)	130 (25)
Patient‐years with ≥2 fractures, *n* (%)	50 (38.4)
Patent years with same site recurrent fractures, *n* (%)	26 (20)
Bisphosphonates	
Patients received bisphosphonates, *n* (%)	21 (47.8)
Median age of starting bisphosphonates, years (IQR)	6.4 (5.0–7.9)
Years when receiving bisphosphonate, *n* (%)	
No bisphosphonate	244 (46.9)
Before bisphosphonate	131 (25.2)
During bisphosphonate	114 (21.9)
After bisphosphonate	31 (6.0)
Fracture rate (per/1000 patient‐years, 95% CI)	
No bisphosphonates	230 (180–280)
Before bisphosphonates	340 (250–420)
During bisphosphonates	210 (130–290)
After bisphosphonates	190 (50–340)

*Note*: Results are number and percentage of total fractures or patient study years.

Of the 43 patients on whom data were available, 35 (80%) had a positive family history of OI and 8 (18%) were index cases. History of gray‐blue sclerae was available for 38 patients and was positive in 36 (95%) patients. Dentinogenesis imperfecta was seen in six patients out of 30 (20%) for whom the information was available. Hypermobile joints were documented in 22 out of 28 patients (78%) where the history was available. All patients were ambulatory at the conclusion of the study period. Of the 44 patients, 21 (48%) received bisphosphonates, totaling 115 (22%) bisphosphonate years.

### Fracture location and frequencies

**Fig. 1:**
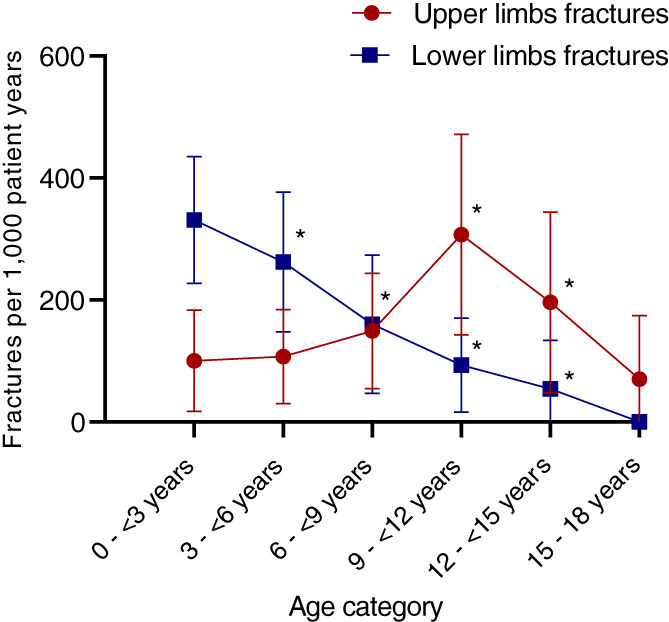
Relationship between age and fracture rate for all fractures. Data show mean and 95% CI by age category.

A total of 197 fractured bones occurred in the 520 patient‐years of the study, giving a mean fracture rate of 379 fractures per 1000 patient‐years (95% CI: 310 to 440) (Table 1). A total of 165 fracture events occurred in 130 patient study years (25% of patient study years). The median age (IQR) of first fracture was 1.4 (0.2 to 2.6) years, and the median age (IQR) for any fracture was 5.0 (2.5 to 9.0) years. Fracture prevalence varied from one or fewer fractures in 22.7% (*n* = 10) of patients to two to 20 fractures in 77.3% (*n* = 34) of patients. The fracture rate was highest in the age group 0 to <3 years (469 fractures/1000 patient‐years), of which 38% occurred in the first year, including three at birth, and the lowest in the 15‐ to 18‐year‐old age group (139 fractures/1000 patient‐years) (Figure 1).

**Fig. 2:**
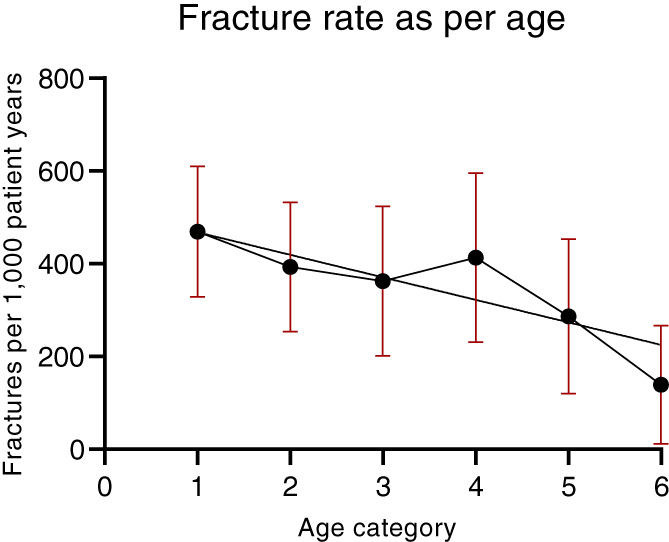
Relationship between age and fracture rate for upper and lower limb fractures. Data are mean and 95% CI by age category. *p < 0.01 compared to group of 0 to <3 years by logistic regression.

Among the long bone fractures, 49.7% were lower limb and 40.6% were upper limb fractures. Neither sex nor family history was related to the occurrence of upper or lower limb fractures. However, the incidence of fractures was strongly related to age (Figure 2). Lower limb fracture was highest in the 0 to <3 years age group at 331 fractures/1000 patient‐years. The incidence of upper limb fractures was very different: 100 fractures/1000 patient‐years in the 0 to <3 years age group and peaking in the 9 to <12 years age group at 307 fractures/1000 patient‐years (Figure 2). The distribution of fracture sites and location is shown in Table 2. Note that the metadiaphyses was identified as the most frequent sites of fracture. Only 25 (14%) were midshaft, while 28 (16%) were at the elbow, 37 (21%) were at the wrist, 19 (11%) were at the knee, and 45 (25%) were at the ankle.

**Table 2:** Fracture location

Fracture location	Proximal, *n*	Middle shaft, *n*	Distal, *n*	Epiphysis, *n*	Not characterized, *n*	Total, *n* (%)	Fractures per 1000 patient‐years
Upper limb							
Humerus	4	2	13		1	20 (25.0)	38
Radius	0	1	27		1	29 (36.2)	56
Ulna	15	4	10		2	31 (38.8)	60
Total upper limb						80 (40.6)	154
Lower limb							
Femur	1	6	5		2	14 (14.3)	27
Tibia	7	10	35	2	6	60 (61.2)	115
Fibula	7	2	10		4	23 (23.5)	44
Total lower limb						98 (49.7)	188
Total long bone						178 (90.3)	342
Axial							
Vertebral						5	
Ribs						1	
Clavicle						7	
Scapula						1	
Other						5	
Total axial						19 (9.7)	36
Total						197 (100)	379

### Prognosis for fracture

To evaluate the effects of age, sex, and family history of OI, these three variables were examined in logistic regression (Table 3). Only age was significant in the model. Patients who avoided fracturing in the first 3 years were not protected from future fracture as 21.2% of those who avoided early fracture developed fractures at a later age compared to 22.9% of those who sustained a fracture at early age (*p* = 0.69 in chi‐squared test).

**Table 3:** Logistic regression of family history, sex, and age for any fracture

Variable	Odds ratio (95%CI)	*p* value
Family history	1.33 (0.81 to2.19)	0.26
Sex	0.89 (0.59 to1.32)	0.55
Age at fracture	0.85 (0.75 to0.97)	<0.01
Constant	0.44	

Out of 197 total fractures, 26 fractures (20% of 130 fracture years) occurred at the same anatomical site as a previous fracture. The time to refracture at the same site varied from 1 month to 5.8 years (mean 2.2 years).

### Bisphosphonates

Twenty‐one (48.5%) patients received bisphosphonates at a median age (IQR) of 6.4 (5.0 to 7.9) years. Prior to commencing antiresorptive therapy, individuals who went on to receive bisphosphonates sustained a fracture in 33% of the years compared to 23% in patients who did not commence bisphosphonates (odds ratio [OR] [95% CI] 1.7 [1.1 to 2.8] *p* = 0.03) (Table 4). During and after bisphosphonate therapy, fracture rates in these patients were similar and not statistically significantly different from the bisphosphonate‐naïve group at 17% and 19% respectively.

**Table 4:** Logistic regression for fracture occurrence in bisphosphonate use categories

Bisphosphonate category	Patient‐years (%)	Fracture years *n* (%)	Odds ratio (95%CI)	*p* value
No bisphosphonate	244 (46.9)	56 (23)	1	
Prior to bisphosphonate	131 (25.2)	44 (34)	1.67 (1.02 to 2.75)	0.04
On bisphosphonate	114 (21.9)	24 (17)	1.09 (0.61 to 1.94)	0.76
After bisphosphonate	31 (6.0)	6 (19)	0.89 (0.34 to 2.39)	0.82
Constant			0.3	

*Note*: Results show number of fracture years and odds ratio for fractures compared to no bisphosphonate patients, adjusted for age, sex, and family history of OI.

## Discussion

### Fracture rates

Our study is the first to report an early age for the peak occurrence of fractures, predominantly in the lower limb compared to the upper limb, that extends the age and limb specific fracture data for OI type 1. A previous Danish study by Folkestad et al. reported on the lifetime fracture incidence in patients with OI, not restricted to OI type 1, which included children from a population‐based cohort of all OI patient categories from the national registry.^[^ ^3^ ^]^ The fracture rate in the age group of 0 to 19 years was 233.9 fractures/1000 patient‐years, somewhat lower than that in our study of 379 fractures/1000 patient‐years in those aged 0 to 18 years, perhaps due to the use of national data for patient detection. Two studies from North America and Canada reported fracture rates similar to those reported by our study.^[^ ^4^, ^5^ ^]^ A recent study from the Netherlands reported that OI patients aged 1 to 19 years experienced the highest hospitalization rate.^[^ ^7^ ^]^

We note that the frequency of lower limb fractures in the younger age group decreased with age. However, upper limb fracture rates increased with age, reaching a peak at 9 to <12 years. This difference in fracture sites may be due to changes in the type of physical activity or mobility and, therefore, to different mechanisms of injury with age; for example, older children are more likely to be involved in sports and therefore have sport‐related injuries and fractures. A study from Sweden on the epidemiology of fractures in children found that the peak age of fractures was 11 to 14 years, with the forearm being the most common site of fractures caused by falls.^[^ ^8^ ^]^ In the early years of life, falls between planes were most common than falls in the same plane and declined significantly by 12 years of age. This may apply to children with OI in relation to fracture site and mechanism but with a higher fracture rate than the general population. The difference in fracture rates between upper and lower limbs in different age groups in children with OI is not well described in the literature. An increased lower limb fracture rate has been reported in conditions with hypermobile joints.^[^ ^9^, ^10^ ^]^ This needs to be explored further in OI. At least half of our cohort had hypermobile joints documented in their records.

Regarding the causation of fractures, during periods of rapid linear growth, the disparity between bone formation and resorption is maximal. Although normal bone has reserve within the bone lineage to increase its rate of matrix formation, the OI bone lineage is already maximally stimulated, so that it is during the period of linear growth that the net bone formation is low.^[^ ^11^ ^]^ This may contribute to the high fracture rates in OI during the periods of rapid growth in childhood, in the first 3 years of life, especially in the first year when growth velocity is at its highest.^[^ ^12^, ^13^ ^]^ A second factor may be gait development, a unique period in which the body displays considerable oscillations due to poor equilibrium^[^ ^14^ ^]^ associated with an increasing risk of falls, which is likely to contribute to the high fracture risk. Besides physiological factors, children and adolescents with OI demonstrate weakness in ankle plantar flexors, have decreased muscle force and power, and possibly decreased postural control from a proprioceptive deficit, all of which could contribute to an increased risk of falls and fractures.^[^ ^15^, ^16^ ^]^

We noted a decline in the fracture rate with increasing age. As growth proceeds, there is an increase in the dimension of the long bones, providing greater structural strength, and there is an accrual of peak lean mass and subsequent bone mass. These changes in bone composition and structure may contribute to a reduction in fracture rates with age.^[^ ^17^, ^18^ ^]^ Fear of fractures and experience of pain at the time of fracture and healing cause reduced activity level.^[^ ^15^ ^]^ This may lead to a learned behavior to avoid situations likely to cause injury and fracture and may contribute to lower fracture incidence with increasing age. Interestingly, the peak incidence for fractures in non‐OI patients appears to coincide with the pubertal growth spurt when there is a relative decrease in size‐corrected bone mineral density due to bone expansion with a relative reduction in bone mass.^[^ ^19^ ^]^

### Same‐site recurrent fractures

The incidence of recurrent fractures in children without OI is low (1.9% to 2.7%) and is predominantly reported in the upper limbs, as found in a study by a major Australian children's bone center in Sydney.^[^ ^20^, ^21^ ^]^ Goulding et al. reported children with an early age of first fracture had higher rates of fracture per l00 years of exposure than those fracturing later.^[^ ^22^ ^]^ We found no studies reporting on same bone site recurrent fractures in the general population of children. In our study, 15% of long bone fractures recurred at the same anatomic site up to three times. Although nonunion of fractures and osteotomies have been reported in patients with OI, fracture healing is generally considered normal in OI.^[^ ^13^, ^23^, ^24^ ^]^ Recent studies on COL1A2‐deficient mice showed a delay in the early phase of OI bone fracture repair possibly due to impaired osteoblastic differentiation of the recruited stem cells.^[^ ^25^ ^]^ An experimental murine study showed that healed fractured tibiae in female OI mice were biomechanically weaker compared with the contralateral nonfractured bone, suggesting that abnormal OI fracture healing in OI may prime future refracture at the same location.^[^ ^26^ ^]^ This is the first paper to report this concern in humans. Further studies on bone healing in OI are required to better understand same‐site recurrent fractures in these patients.

### Bisphosphonates

Our study reports that children with OI type 1 at high fracture risk who commenced bisphosphonate treatment subsequently had a one‐third lower yearly fracture risk, similar to that of the low fracture risk group that did not receive bisphosphonates. Because of the observational design of the study, these findings remain uncertain but, if correct, identify a substantial reduction in fractures. These findings are consistent with previous data in a systematic review of bisphosphonate therapy in children with OI, in which most studies reported fracture reduction over 2 years of therapy.^[^ ^27^ ^]^

### Limitations

We report only the long bone and axial fractures for which patients attended hospital for care. This did not include short bone fractures of hands and feet, which can also lead to a loss of function and mobility. Since this was a retrospective study, the actual fracture rate may be higher than reported as some patients may not have accessed the tertiary center for all fractures, some of which might have been treated at home or without radiological confirmation. Vertebral fractures are frequent in patients with OI; however, we report a low incidence of vertebral fractures compared to the literature.^[^ ^5^ ^]^ This is likely because we only captured symptomatic fractures as regular surveillance with spine X‐rays to capture asymptomatic vertebral fractures was not part of the routine clinical care for our patients with OI during the study period. Bisphosphonate therapy was recorded as years of therapy and did not consider the exact dose or type of intravenous bisphosphonates.

In conclusion, the presented data confirm the importance of young age in the incidence of fracture on OI type 1 patients, especially in fractures of the lower limb, highlighting important differences with respect to non‐OI patients in fracture pattern and fracture burden. These findings warrant prompt evaluation of fracture treatment and postfracture rehabilitation in children with OI early in life. Current guidelines do not emphasize the importance of physiotherapy in early childhood development.^[^ ^9^ ^]^

A multidisciplinary team approach should be implemented for the overall health care of children with OI. This includes specialized clinicians, physiotherapists, and occupational therapists to address the functional and motor areas of children with OI, social workers to navigate support services, and specialized nurses for coordinating patient care. First responders, such as general practitioners, paramedics, and emergency service staff, should be educated about the increased risk of fractures in children with OI so that they can appropriately triage and provide prompt management of fractures.

## Author Contributions

**Kiranjit K Joshi:** Conceptualization; investigation; writing – original draft; writing – review and editing; methodology; data curation. **Aris Siafarikas:** Conceptualization; writing – review and editing; supervision. **Richard Prince:** writing – review and editing.

### Peer Review

The peer review history for this article is available at https://www.webofscience.com/api/gateway/wos/peer-review/10.1002/jbm4.10782.
